# Development and validation of the Oral health behavior questionnaire for adolescents based on the health belief model (OHBQAHBM)

**DOI:** 10.1186/s12889-020-08851-x

**Published:** 2020-05-15

**Authors:** Bilu Xiang, Hai Ming Wong, Wangnan Cao, Antonio P. Perfecto, Colman P. J. McGrath

**Affiliations:** 1 Department of Paediatric Dentistry, Faculty of Dentistry, The University of Hong Kong, 2/F The Prince Philip Dental Hospital, 34 Hospital Road, Sai Ying Pun, Hong Kong, SAR China; 2 Department of Dental Public Health, Faculty of Dentistry, The University of Hong Kong, Hong Kong, SAR China

**Keywords:** Questionnaires, Adolescents, Oral health-related behavior, Validation, Psychological factor

## Abstract

**Background:**

Oral health belief is a prerequisite of changing oral health behaviors especially during adolescence. However, there is a paucity of well-established questionnaire for use among adolescents. This study aimed to develop and validate an instrument to evaluate adolescents’ beliefs about oral health behaviors using health belief model.

**Methods:**

A preliminary 43-item questionnaire was developed by an expert panel. Then the questionnaire was finalized by decreasing the number of items to 35 by analyzing the results from face validity and factor analysis from 421 Hong Kong secondary school students. The content validity were evaluated by a panel of 2 behavioral scientists, 2 dentists, 2 schoolteachers and 10 adolescents. The construct validity of the questionnaire was assessed by performing exploratory factor analysis (EFA) and confirmatory factor analysis (CFA). The Cronbach’s alpha coefficient, item-total correlation and intraclass coefficient were used to test its reliability. In addition, to confirm its applicability, multiple regression analysis and path analysis were used to evaluate the possibility of HBM as predictors for oral health behaviors and oral hygiene status.

**Results:**

The initial analysis extracted six factors that jointly accounted for 62.47% of the variance observed. Based on CFA, the final version of the questionnaire consisted of 35 items and the data of the final version fitted the model well. The Cronbach’s alpha coefficient for the subscale (> 0.7), item-total correlations (0.47–0.91) and the intraclass coefficient (0.82–0.91) were all above acceptable thresholds. The results of multiple regression analysis and path analysis confirmed its ability to predict oral health behaviors and status.

**Conclusions:**

The present findings indicate satisfactory validity, reliability and applicability of the proposed Oral Health Behavior Questionnaire for Adolescents based on the Health Belief Model (OHBQAHBM) for measuring oral health beliefs of adolescents. This questionnaire can be used as an instrument to measure oral health beliefs and predict oral health behavior and oral hygiene status of adolescents.

## Background

Oral health is an integral part of general health, which influences various aspects of life, such as chewing, speaking, appearance and socializing [1, 2]. Dental complications can result in the absence from school and poorer academic performance [3]. Oral diseases are major public health problems, which rank as the fourth most expensive diseases in developed countries [2]. In some countries, the expenditure on dental caries of children alone exceeds the total budget for children’s oral health care [4]. Some preventive measures, such as water fluoridation, help to reduce the occurrence of caries, but inadvertently increase the prevalence and severity of fluorosis [5]. Adolescence is a formative period for young people to develop their adult lifestyles, including oral health behaviors [6]. However, inadequate attention and guidance have been given for dental health care [7], especially in Hong Kong. For instance, the use of dental floss and annual dental visitations among Hong Kong adolescents remain relatively unimportant [8].

Multiple theories have long been applied to explain the psychological determinants of behavior and health promotion [9]. The health belief model (HBM), consisting of six main concepts: perceived susceptibility, perceived benefits, perceived severity, perceived barriers, cues to action and self-efficacy, is one of the most widespread behavioral science models [10, 11]. Based on the HBM, oral health belief has been shown to be a prerequisite of changing oral health behaviors [12]. Several studies have confirmed the applicability and effectiveness of HBM in predicting oral health behaviors [11, 13]. No previous standard instrument has been validated to assess adolescents’ beliefs towards oral health behaviors. In order to provide a more targeted oral health service plan specifically designed for adolescents, it is important to collect information about their attitudes and beliefs towards oral health behaviors. Therefore, this study aimed at developing and validating a specific instrument, the Oral Health Behavior Questionnaire for Adolescents based on the Health Belief Model (OHBQAHBM), which measures factors affecting oral health behaviors of adolescents based on the HBM. In addition, we tried to describe the relationship among oral health beliefs, oral health behaviors and oral hygiene status.

## Methods

### Ethical considerations

The study was approved by the HKU/HA HKW Institutional Review Board (IRB HKU: UW17–348). Written consents from the parents were obtained and confidentiality of their privacy was assured. The rights of participants to withdraw at any time were guaranteed.

#### Phase I: development and pilot test of the questionnaire

##### Literature searching and drafting

Prior to the study, a literature search was performed between April and June 2018 to identify instruments that adopted HBM components within an oral health context. The Cochrane Library, PubMed, and Google Scholar databases were searched using keywords: oral health, health belief model/ HBM, adolescent, child, schoolchildren, validation and development of instruments. We did not identify any validated oral health-related instruments based on HBM specifically designed for adolescents. Three researchers (one with extensive experience in the development of psychometric questionnaires, one with professional knowledge in adolescence health behaviors, and the other Specialist in Pediatric Dentistry) drafted the questionnaire after referring to the questionnaires utilized in similar contexts [14–16]. The drafted questionnaire containing 43 items was sent to a linguistics expert for verification of clarity, conciseness and grammar.

##### Face and content validity

A panel of 2 behavioral scientists, 2 dentists and 2 schoolteachers were invited to investigate the relevance and conceptual scope of the items in relation to HBM. After discussion, all panel members agreed with some minor modifications and to delete one item from the questionnaire. The revised questionnaire was pilot-tested with a convenience sample of 10 adolescents (12–17 y) to ensure the clarity of the questionnaire. The time required to complete the questionnaires was approximately 15–20 min for each individual. These adolescents were encouraged to ask any question and make comments to any part of the questionnaire. All comments and suggestions were considered to omit potential misunderstandings. The initial version was modified and confirmed by the discussions of expert panel and comments from voluntary adolescents. It contained 42 items in six subscales which include: Perceived Susceptibility (SUS 1–2), Perceived Benefits (BEN 3–10), Perceived Barriers (BAR 11–19), Cues to Action (CUE 20–23), Perceived Severity (SEV 24–32), Self-efficacy (EFF 33–42). A five-point Likert format was adopted in the options of answers. The items of SUS, BEN, BAR and CUE have the following response options: strongly disagree (scores 1 point), disagree (scores 2 points), neutral (scores 3 points), agree (scores 4 points) and strongly agree (scores 5 points). The items of SEV have response choices: not serious (scores 1 point), a little serious (scores 2 points), partially serious (scores 3 points), serious (scores 4 points) and very serious (scores 5 points). The items of EFF include five choices of answers: not confident (scores 1 point), a bit confident (scores 2 points), fairly confident (scores 3 points), quite confident (scores 4 points) and very confident (scores 5 points). The average score of each subscale was calculated to represent the individual’s belief towards that specific domain. For each subscale, higher scores indicate stronger feelings towards each domain.

#### Phase II: item reduction and questionnaire testing

##### Questionnaire structure

The version-1 of the questionnaire consisted of the following three sections. The first part contained the demographic information (age and gender); the second part included the 42-item structured questionnaire about the constructs of HBM; the third part consisted of three questions concerning oral health behaviors. In the third part, respondents were asked to report the frequency of tooth brushing (1. Less than twice a day; 2. Twice or more a day), flossing frequency (1. Less than once a week or never; 2. Once or more a week) and dental visits (1. No regular dental visit; 2. Have an annual dental visit). Data were collected from July to September 2018. In order to perform test-retest reliability analysis, 40 adolescents from the sample (*n* = 421) were randomly selected to complete the questionnaire twice within a two-week interval.

##### Sampling and data collection

The version-1 of the questionnaire was administered to adolescents in Hong Kong. The sample size was determined by referencing the requirements of explanatory factor analysis (EFA). As a general rule of thumb, a subject-to-item ratio of 1:10 was adopted [17]. At last, the sample size was calculated as 420. Each secondary school in Hong Kong was assigned a number (1 to 113) and three schools were randomly selected from a random numbers table. Afterward, all S2 students of these three schools were assigned a number and 470 students were invited to participate by selecting from the random numbers table, with an expected 90% response rate. Participants undergoing orthodontic treatments were excluded. Finally, 421 adolescents were eligible and agreed to participate in the study. The questionnaire was self-administered and the whole process was monitored by a researcher and a schoolteacher. If anyone did not understand the questions, he or she could ask the researcher.

##### Clinical measurements

The visual plaque index (VPI) score [18] was used to evaluate plaque accommodation at buccal surfaces of teeth. An index for the entire mouth is determined as dividing the total score by the number of surfaces examined. Two trained and calibrated dentists conducted the dental examination in schools. Calibration was executed on a separate sample of adolescents (*n* = 27, 11 boys and 16 girls) prior to the study. The weighted kappa coefficient value for inter-examiner reliability was 0.87. Then the dentists rated the same adolescents for VPI 1 h later to determine intra-examiner reliability. The weighted kappa coefficient value was 0.94 for examiner 1 and 0.88 for examiner 2. Both examiners showed good reliability.

##### Construct validity for item reduction

SPSS 25.0 and AMOS 22.0 were utilized to analyze data. Construct validity was examined using EFA and confirmatory factor analysis (CFA). The principal component analysis with a varimax rotation was applied to extract factors. Kaiser-Mayer-Olkin (KMO) > 0.6 and Bartlett’s test for sphericity (*P* <  0.05) were considered as sampling adequacy. Parallel analysis was conducted for factor extraction [17]. The extracted factors were rotated orthogonally using the varimax procedure. The acceptable level of factor loading was set at 0.3 and above [19, 20]. CFA was performed to evaluate the coherence between the data and the structure model. The model fit was evaluated using multiple fit indices, including the chi-square statistics (χ2); normed chi-square (χ2/ df); comparative fit index (CFI); Tucker-Lewis index (TLI); and root mean square error of approximation (RMSEA). CFI > 0.90, TLI > 0.90, RMSEA and SRMR < 0.06 indicated a good fit (SRMR < 0.08 acceptable) [21, 22]. The indicators that are conceptually more distant from the supposed latent variable result in a lower loading (< 0.5) and should be removed [13].

#### Phase III: practicability of the questionnaire

##### Reliability and stability

The reliability of the final-version questionnaire was evaluated using item-total subscale correlations and Cronbach’s alpha coefficients. A correlation of < 0.30 between an item and the total subscale score was considered poorly functioned [20]. A value of 0.70 or above of Cronbach’s alpha was considered evidence of internal consistency [23]. Stability was measured by performing test-retest reliability analysis via intraclass correlation coefficients (ICC). Values of ICC between 0.75 and 0.90, and greater than 0.90 indicate good and excellent stability, respectively [24].

##### Criterion validity for predicting oral health behaviors

To explore the criterion-oriented validity of the final-version questionnaire, the association between desirable oral health behaviors (brushing twice or more a day, floss weekly or more, and regular dental visit) and HBM components including perceived susceptibility, perceived benefits, perceived barriers, perceived severity, cues to action and self-efficacy was evaluated by performing multiple logistic regression analysis.

##### Structure equation modeling for predicting oral hygiene status

**Fig. 1:**
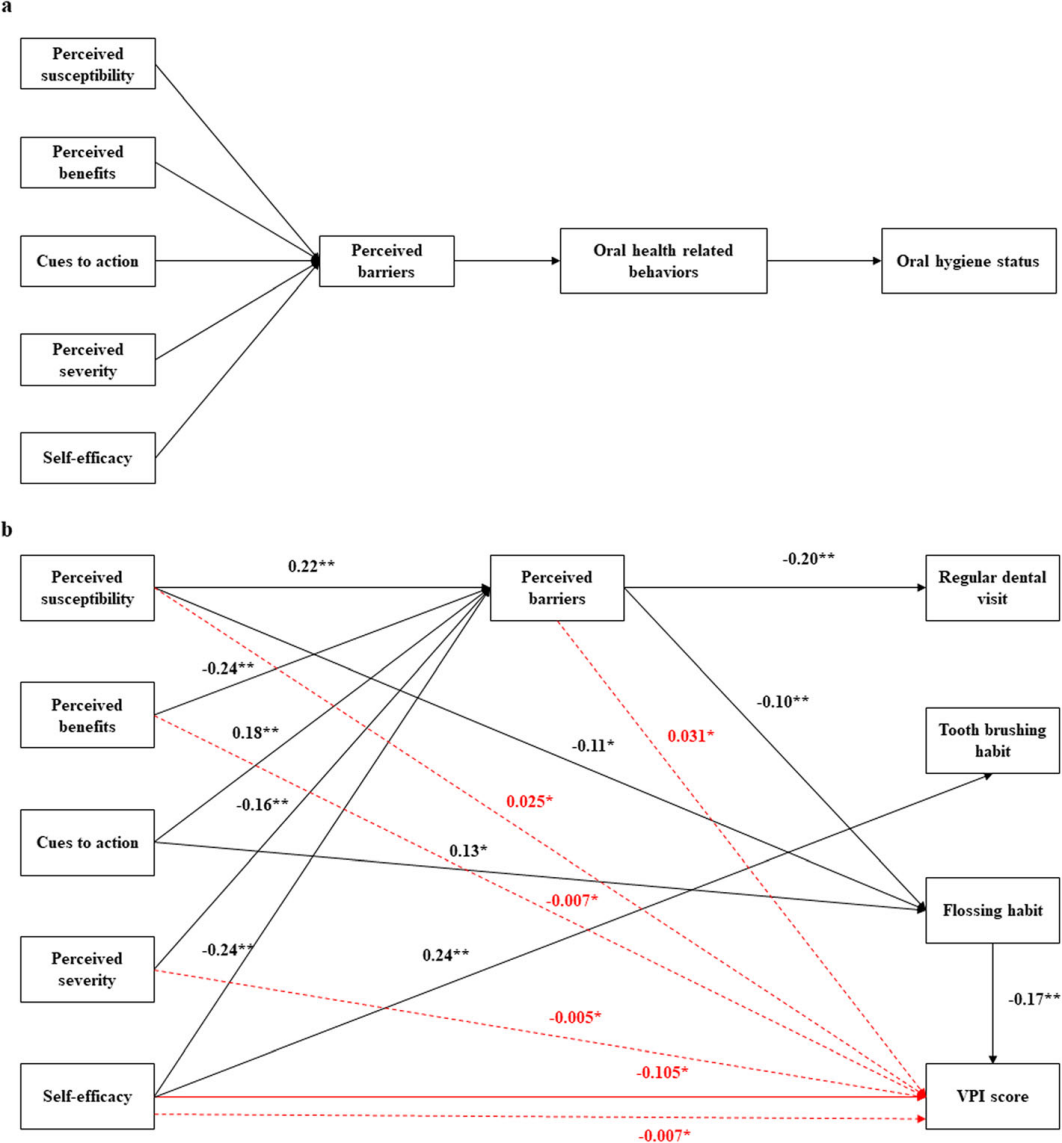
**a**. A theoretical model of the impact of HBM on VPI score through oral health behaviors. **b**. The final model of path analysis. Standardized direct and indirect effects of HBM variables on VPI score were represented with solid and dotted red lines, respectively (*p < 0.05, **p < 0.001). Error terms and covariance are not presented for ease of understanding

Path analysis was utilized to assess the causal relationship between HBM beliefs, behaviors and oral hygiene status in AMOS 22.0. Based on the suggestion of previous investigations, perceived barriers might play the most important role in predicting behaviors in HBM [25, 26]. It was hypothesized in our model that perceived benefits, perceived severity, perceived susceptibility, self-efficacy and cues to action might influence behaviors through barriers. And all the beliefs would have a direct effect on oral health behaviors, which in turn would influence the oral hygiene status of adolescents (Fig. 1a). Tooth brushing, flossing habits and dental visit patterns were converted to dummy variables (1 = undesirable behaviors, 2 = desirable behaviors: brushing twice or more a day, flossing once a week or more, have an annual dental visit). Other variables in the model were used as continuous variables. Direct and indirect effects on VPI were estimated using bootstrap with bias-corrected 95% confidence intervals. The degree of correspondence between the conceptual model and actual data was evaluated using a good-of-fit test. The cut-off criteria to consider the model a good fit to the data included CFI > 0.90, TLI > 0.90, RMSEA and a standardized root mean square residual (SRMR) <  0.06 [22].

## Results

### Phase II: item reduction and questionnaire testing

#### EFA results

In total, 421 secondary school students were entered into the study. The KMO value was 0.88 and Bartlett’s test was<0.001, which was adequate for EFA. Table 1 shows that six factors were extracted by using rotated factor analysis. The six factors jointly accounted for 62.49% variance observed. Factor 1 accounted for 24.79% of the variance, representing all 10 items of Self-efficacy scale. Factor 2 accounted for 11.91% of the variance and represented all items of Perceived benefits scale. The items of Perceived severity subscale were loaded together as factor 3 and accounted for 9.27% of the variance. Factor 4 accounted for 7.33% of the variance and represented items regarding the potential barriers. Factor 5 accounted for 5.21% of the variance and represented the cues to take action. Factor 6 accounted for 3.98% of the variance and represented the remaining two items regarding susceptibility (Table 1).

**Table 1:** Rotated factor analysis of Health Belief Model Scale for oral health behaviors

Factor 1 Self-efficacy		Factor 2 Perceived benefits		Factor 3 Perceived severity		Factor 4 Perceived barriers		Factor 5 Cues to action		Factor 6 Perceived susceptibility	
EFF37	0.92	BEN5	0.83	SEV27	0.79	BAR14	0.84	CUE22	0.88	SUS1	0.77
EFF38	0.90	BEN6	0.81	SEV26	0.77	BAR13	0.82	CUE21	0.86	SUS2	0.75
EFF34	0.90	BEN7	0.80	SEV25	0.74	BAR19	0.56	CUE20	0.75		
EFF39	0.88	BEN3	0.78	SEV30	0.71	BAR15	0.55	CUE23	0.43		
EFF33	0.88	BEN9	0.74	SEV28	0.71	BAR12	0.53				
EFF42	0.87	BEN8	0.70	SEV24	0.68	BAR16	0.51				
EFF40	0.86	BEN10	0.70	SEV29	0.67	BAR17	0.50				
EFF41	0.86	BEN4	0.44	SEV31	0.55	BAR18	0.49				
EFF35	0.83			SEV32	0.55	BAR11	0.32				
EFF36	0.82										
Eigenvalue											
10.41		5.00		3.89		3.08		2.19		1.67	
Variance explained											
24.79		11.91		9.27		7.33		5.21		3.98	

#### CFA results

**Fig. 2:**
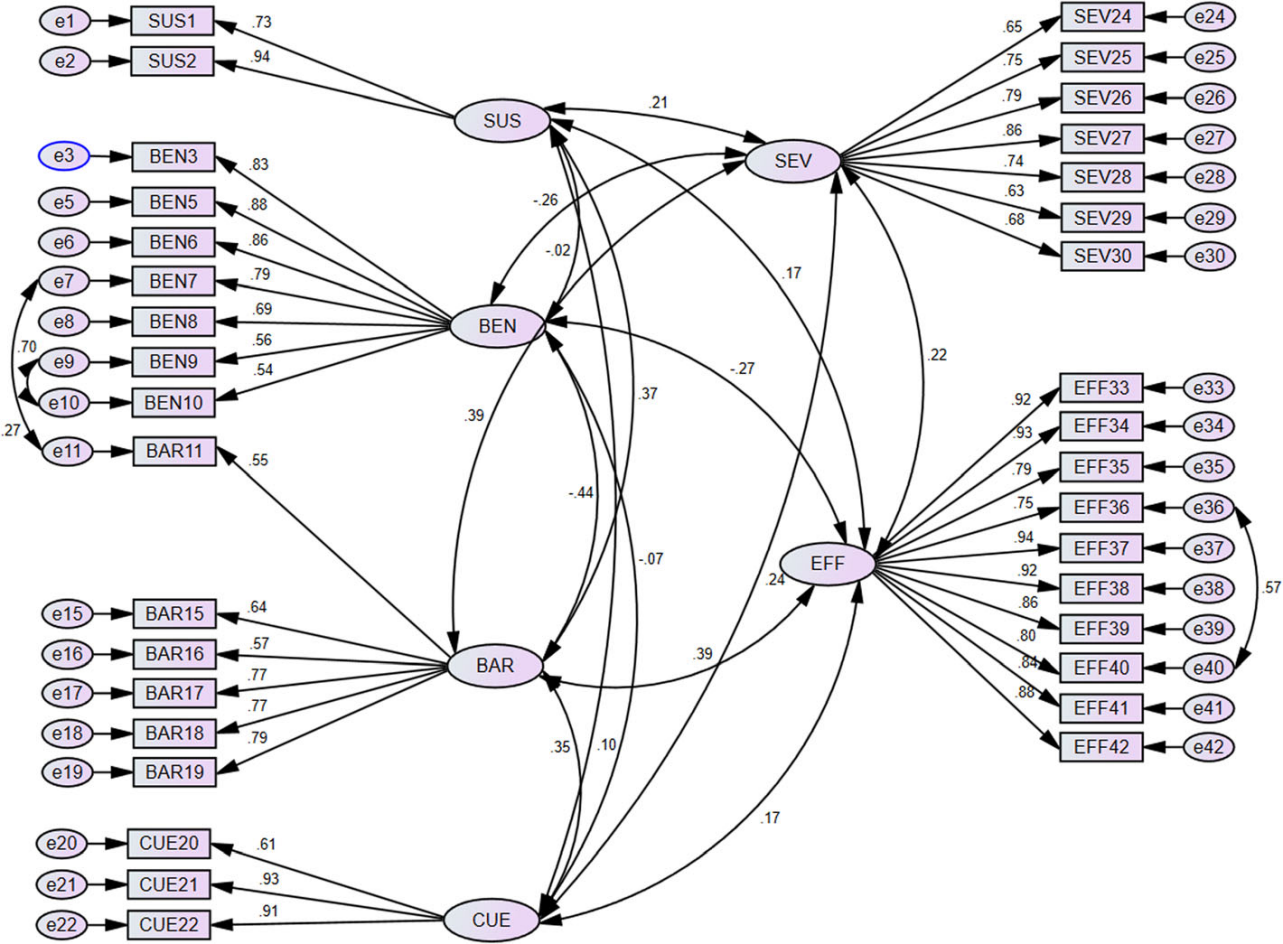
The final version of 35-item OHBQAHBM following CFA of 421 adolescent data fitted the Health Belief Model and its constructs. Standardized coefficients were shown with black lines

CFA was used to test whether the pattern of relationships among the items could be explained by the six-factor model extracted by EFA. At first, the measurement model did not fit the data. Overall, the loading factors of 7 items (BEN4, BAR12, BAR13, BAR14, CUE23, SEV31 and SEV32) were <  0.5 and were omitted from the final version. The loading factors of the remaining 35 items were all> 0.5 and should be considered important [27]. In addition, some correlations between the variables’ errors were added to the model based on the modification indices. After the modification, the fit indices for the final 35-item model were χ2 = 1415.32, df = 543, *P* <  0.001, CFI = 0.92, TLI = 0.91, RMSEA = 0.062 (90% CI 0.058 to 0.066), indicating an acceptable fit to the data (Fig. 2).

### Phase III: practicability of the questionnaire

The mean age of participants was 13.2 (SD = 0.5) years old and 50.1% of them were girls. Most of the adolescents brushed twice or more a day (80.8%), had dental floss less than once a week (77.0%) and did not have a regular dental visit plan (65.8%). The mean subscale scores of the final-version questionnaire were 2.6 ± 0.9 for perceived susceptibility, 4.0 ± 0.6 for perceived benefits, 2.1 ± 0.7 for perceived barriers, 2.1 ± 0.9 for cues to action, 3.8 ± 0.8 for perceived severity, and 3.6 ± 1.0 for self-efficacy. The mean VPI score was 2.3 ± 0.6 (Additional file 1: Table S1).

#### Reliability and stability

After item reduction, we tested the reliability and stability of the final 35-item instruments. Score means, standard deviations, item-total correlations and Cronbach’s alpha if item deleted for each question were listed in Table 2. Each items’ item-total correlations were above 0.30 and Cronbach’s alpha coefficient of the six subscales ranged from 0.81–0.97 (Table 2). The intraclass coefficients ranged from 0.82 to 0.91, demonstrating a good stability (Table 3).

**Table 2:** Item-total correlation and internal consistency of Health Belief Model Scale for oral health behaviors

Items of subscale		Mean	SD	Item-total correlation	Cronbach’s α If item deleted (*n* = 421)	Cronbach’s α of the subscale (*n* = 421)	Test-retest intra-class correlation (*n* = 40)
Subscale of perceived susceptibility						0.81	0.82
SUS1	There is a chance that I will get caries.	2.73	1.00	0.69	–		
SUS2	There is a chance that I will have periodontal disease.	2.47	0.95	0.69	–		
Subscale of perceived benefits						0.90	0.80
BEN3	I think brushing and flossing can make teeth healthier.	4.13	0.77	0.73	0.88		
BEN5	I think brushing and flossing can prevent oral diseases from happening.	4.14	0.76	0.78	0.87		
BEN6	I think brushing and flossing can make teeth look good.	4.09	0.73	0.77	0.88		
BEN7	I think brushing and flossing can keep breath fresh.	4.02	0.81	0.72	0.88		
BEN8	I think brushing and flossing can prevent inconvenient eating caused by oral diseases.	4.11	0.78	0.66	0.89		
BEN9	I think brushing and flossing can help me avoid spending more time on dental treatment in the future.	3.81	0.84	0.66	0.89		
BEN10	I think brushing and flossing can help me avoid spending more money on dental treatment in the future.	3.78	0.91	0.62	0.89		
Subscale of perceived barriers						0.84	0.88
BAR11	I think it is difficult for me to brush twice a day.	1.83	0.92	0.47	0.83		
BAR15	I think it’s a waste of time to brush and floss.	1.99	0.90	0.59	0.81		
BAR16	I think I do not have enough time to have an annual dental visit.	2.55	1.09	0.54	0.82		
BAR17	I am afraid of undergoing tooth treatment, so I don’t have dental visits annually.	1.93	0.89	0.68	0.79		
BAR18	I think we have no money at my home, so I don’t have an annual dental visit.	1.94	0.97	0.68	0.79		
BAR19	I think the dental clinic is far from my home, so I don’t have an annual dental visit.	2.06	1.01	0.71	0.78		
Subscale pf cues to action						0.85	0.85
CUE20	Parents often reminds me of brushing and flossing	2.48	1.13	0.58	0.92		
CUE21	Classmates often reminds me of brushing and flossing.	1.87	1.02	0.79	0.72		
CUE22	Teachers often reminds me of brushing and flossing.	1.95	1.04	0.79	0.72		
Subscale of perceived severity						0.89	0.88
SEV24	If I have caries, for me that is …	3.36	1.05	0.61	0.88		
SEV25	If I have periodontal disease, for me that is …	3.86	1.02	0.71	0.87		
SEV26	If my teeth do not look good because of oral diseases, for me that is …	3.60	1.02	0.72	0.87		
SEV27	If I have bad breath because of oral diseases, for me that is …	3.80	0.98	0.79	0.86		
SEV28	If I can’t sleep well because of oral diseases, for me that is …	1.06	1.02	0.69	0.87		
SEV29	If I can’t eat my favorite food because of oral diseases, for me that is …	4.12	1.05	0.60	0.88		
SEV30	If I get laughed at by classmates because of oral diseases, for me that is …	3.82	1.07	0.64	0.88		
Subscale of self-efficacy						0.97	0.91
EFF	How confident you are that you will brush your teeth for 2 min twice daily on the circumstances below?						
EFF33	When you are under a lot of stress	3.77	1.04	0.87	0.96		
EFF34	During or after experiencing personal problems	3.74	1.08	0.88	0.96		
EFF35	When you are feeling tired	3.44	1.15	0.80	0.97		
EFF36	When you don’t feel like it	3.35	1.20	0.79	0.97		
EFF37	When you are anxious	3.78	1.05	0.91	0.96		
EFF38	After experiencing family problems	3.72	1.11	0.88	0.96		
EFF39	When you have other commitments	3.71	1.08	0.87	0.96		
EFF40	When you feel you don’t have the time	3.49	1.18	0.84	0.97		
EFF41	When you are feeling under pressure from school work	3.69	1.16	0.84	0.97		
EFF42	When you have too much work to do at home	3.80	1.05	0.86	0.96

**Table 3:** HBM factors for predicting favorable oral health behaviors

	B	SE	Wald statistics	OR (95% CI)	P
Predicting brushing twice daily (*N* = 412, Cox & Snell R^2^ = 0.097, Nagelkerke R^2^ = 0.160) Chi-square = 42.2, df = 6, *p* < 0.001					
Perceived susceptibility	−0.16	0.17	0.91	0.85 (0.61–1.19)	0.34
Perceived benefits	0.14	0.25	0.31	1.14 (0.71–1.86)	0.58
Perceived barriers	−0.10	0.25	0.16	0.91 (0.56–1.47)	0.69
Cues to action	0.21	0.18	1.43	1.23 (0.88–1.74)	1.23
Perceived severity	0.14	0.20	0.48	1.15 (0.78–1.69)	0.49
Self-efficacy	0.86	0.17	25.98	2.36 (1.70–3.28)	< 0.001
Predicting weekly flossing behavior (*N* = 418, Cox & Snell R^2^ = 0.069, Nagelkerke R^2^ = 0.106) Chi-square = 30.0, df = 6, *p* < 0.001					
Perceived susceptibility	−0.30	0.14	4.42	0.74 (0.56–0.98)	0.04
Perceived benefits	−0.01	0.22	0.001	0.99 (0.65–1.53)	0.98
Perceived barriers	−0.86	0.24	12.74	0.42 (0.26–0.68)	< 0.001
Cues to action	0.31	0.14	5.26	1.36 (1.05–1.77)	0.02
Perceived severity	−0.10	0.17	0.37	0.90 (0.65–1.26)	0.54
Self-efficacy	−0.02	1.14	0.01	0.98 (0.75–1.29)	0.91
Predicting regular dental visit (*N* = 419, Cox & Snell R^2^ = 0.070, Nagelkerke R^2^ = 0.098) Chi-square = 30.6, df = 6, *p* < 0.001					
Perceived susceptibility	0.09	0.13	0.53	1.10 (0.86–1.41)	0.47
Perceived benefits	−0.21	0.19	1.15	0.81 (0.56–1.19)	0.28
Perceived barriers	−1.10	0.22	26.14	0.33 (0.22–0.51)	< 0.001
Cues to action	0.08	0.12	0.37	1.08 (0.85–1.37)	0.54
Perceived severity	−0.23	0.15	2.25	0.80 (0.59–1.07)	0.13
Self-efficacy	−0.16	0.12	1.71	0.85 (0.67–1.08)	0.19

#### Criterion validity for predicting oral health behaviors

The criterion validity was determined by evaluating the association between desirable oral health behaviors and HBM components. The logistic regression analysis was performed respectively for each dependent outcome variables (brushing, flossing and dental visit behavior). The independent variables, including perceived susceptibility, perceived barriers, perceived benefits, cues to action, perceived severity and self-efficacy, were entered into the model. The results are shown in Table 3. The outliers were excluded based on Cook’s distances and standardized residuals. For predicting brushing behaviors, 412 cases were entered into the model and the full model was statistically reliable (χ2 = 42.2, df = 6, *p* < 0.001). This model explained 9.7 to 16% of the variance. The results showed that self-efficacy had a positive effect on brushing twice or more a day (OR = 2.36, 95% CI = 1.70–3.28, *p* < 0.001). In addition, 418 adolescents were entered in the analysis to predict flossing behaviors (χ2 = 30.0, df = 6, *p* < 0.001). This model explained 6.9–10.6% of the variance for weekly flossing. Perceived susceptibility (OR = 0.74, 95% CI = 0.56–0.98, *p* = 0.04) and perceived barriers (OR = 0.42, 95% CI = 0.26–0.68, *p* < 0.001) had a preventive effect on flossing habit. On the other hand, cues to action (OR = 1.36, 95% CI = 1.05–1.77, *p* = 0.02) increased the chance of performing a weekly flossing habit. Finally, 419 individuals were included in the full model to predict regular dental visit plans (Cox & Snell R2 = 0.070, Nagelkerke R2 = 0.098, χ2 = 30.6, df = 6, *p* < 0.001). Perceived barriers had a preventive effect on having dental visit plans (OR = 0.33, 95% CI = 0.22–0.51, *p* < 0.001). To conclude, the components of HBM was statistically associated with oral health behaviors, suggesting criterion-oriented validity. In addition, HBM variables might function as predictors for oral health behaviors.

#### Path analysis for predicting oral hygiene status

Based on the conceptual model, we developed an initial model involving all the variables that directly or indirectly affected VPI score which fit with the hypothesized model (TLI = 0.960; CFI = 0.993; RMSEA = 0.027; SRMR = 0.020; χ2 = 6.510; df = 5; *p* = 0.260). After deleting some insignificant paths, the final model was developed (Fig. 1b) and the goodness of model fit improved (TLI = 1.042; CFI = 1.000; RMSEA< 0.001; SRMR = 0.025; χ2 = 15.769; df = 23; *p* = 0.865). Figure 1b demonstrated that self-efficacy had a direct effect on VPI score (β = − 0.105, *p* = 0.015, Additional file 2: Table S2). In addition, perceived susceptibility, perceived benefits, perceived severity, self-efficacy and perceived barriers all had significant indirect effects on VPI score (*p* < 0.05).

## Discussion

The present study developed and validated an instrument for assessing factors related to oral health contexts for adolescents based on the HBM. To our knowledge, OHBQAHBM is the first validated HBM-based questionnaire for adolescents’ oral health. The HBM was first developed in the 1950s and has been applied to a wide range of health behavior promotions [28]. This model emphasized two aspects of the individual’s belief of health behaviors: threat perception and behavioral evaluation [29]. For health problem prevention, the individual should first feel personally susceptible to particular health issues (perceive susceptibility), be able to anticipate the potential severity of the illness (perceived severity), believe in the benefits of performing recommended health behaviors (perceived benefits) and be able to overcome the costs of enacting that particular behavior (perceived barriers) [28]. In addition, cues to action can activate health behaviors when self-efficacy is established [29, 30]. Fatemeh et al. found that HBM was able to predict oral health behaviors and the reduction of perceived barriers could help promote oral health behaviors [11]. With an increase in an individual’s positive perceptions of HBM components, correct brushing and flossing practices were encouraged [31, 32]. Furthermore, one study reported a correlation between the increasing perceived severity and a decrease of DMFT [31]. Therefore, the establishment of healthy oral behaviors in adolescence was essential because growth and development were highly influenced by the effects of oral diseases [33, 34]. Dramatic improvement in adolescent oral health behaviors was optimized when changes in health beliefs occur; therefore developing a standard instrument to assess the beliefs of oral health behaviors for this specific age group was targeted.

In this study, self-administration and anonymity of questionnaires were used to increase respondent’s willingness to disclose sensitive information. The researcher could not influence the respondent’s answers by using her voice or facial expression to imply that a particular answer is the “right” one. Therefore, the interviewer bias was not introduced in the survey results. The construct validity was assessed to test the degree of data fit with the model by performing EFA and CFA. After deleting seven items, CFA indicated acceptable fit indices of the remaining 35 items for the six-domain constructual model. Then, the reliability and practicability of the final 35-item questionnaire were tested. The internal consistency reliability was strong for the overall scale as well as each subscale. The test-retest reliability and item-total correlation analysis were also assured, which further confirmed the practicability of the questionnaire.

Previous research has identified self-efficacy and perceived barriers as a predictor of toothbrushing and flossing behaviors [35, 36]. Similarly, in our study, perceived barriers could serve as a predictor for flossing and dental visit patterns while self-efficacy predicted toothbrushing behavior. In Hong Kong, only schoolchildren from elementary schools are eligible for a free annual dental check-up. Adolescents are at a risk for late diagnosis and treatment of oral diseases due to the discontinuity of free dental check-up services provided by Hong Kong government. In our study, 65.8% of adolescents did not have a regular dental visit plan. The question items of perceived barriers related to dental visit include: 1) do not have enough time (Mean = 2.55); 2) afraid of undergoing tooth treatment (Mean = 1.93); 3) have no money at my home (Mean = 1.94); 4) the dental clinic is far from my home (Mean = 2.06). The only reason due to themselves (afraid of undergoing tooth treatment) got the lowest score. However, family support usually cannot be modified, but the barriers of themselves can be overcome through their endeavor. Moreover, perceived susceptibility and cues to action were significant factors for predicting flossing habit. It is interesting that there was no direct relationship between perceived severity/ benefits and oral health behaviors, which is consistent with the previous study [36]. This result is contrary to the logic that one must feel threatened (perceived severity) and believe that the outcome of behavior change is favorable (perceived benefits) before one intends to change one’s behavior. The possible explanation was that these two components influenced behavior change in a more complex way. For example, one study found that perceived severity was more effective when self-efficacy was higher [37]. On the other hand, perceived benefits might play a role at a later stage when self-efficacy and severity were high [25]. On the whole, the HBM variables couldn’t perfectly explain the variance of oral health-related behaviors. It might be due to the reason that the oral health behaviors recorded in our study were not exhaustive. Detailed information such as the technique of brushing and types of toothbrush may need to be collected in future studies.

To further explore the direct and indirect effect of HBM components on oral hygiene status, the path analysis was conducted. Previous research has tried to refine the model and the perceived barriers have been identified as a strong predictor for behaviors [38]. In fact, the dominant effect of perceived barriers on oral health status has also been confirmed [31]. The unique status of perceived barriers might be derived from the fact that it focused on the current problems for adopting a behavior, rather than a perception of possible future outcomes. Accordingly, the other five components had a significant direct effect on perceived barriers based on our results. Self-efficacy was the only factor identified in our model that had a direct effect on VPI score. Bacterial plaque is the direct cause of gingivitis and adult periodontitis, as well as dental caries [39]. VPI is an indicator of bacterial load and can act as a risk factor of oral diseases. This result was in accordance with the findings of Mizutani that self-efficacy had a direct effect on gingival health [40]. It indicated that the decision to perform oral health practices was influenced by the confidence of doing it successfully. In the present study, except for cues to action, other factors were all found to have a significant indirect effect on oral hygiene. The possible reason might be that cues to action remained undeveloped in the whole framework of HBM and this study only evaluated the external cues. However, a meta-analysis pointed out that the size of the effects for HBM components varied if the targeted behavior differed [38]. In addition, their effects on behavior might also be moderated by each other [41]. Therefore, the theoretical path of HBM’s effect on behaviors and health outcomes is not understood by now due to its complexity and interactions within HBM components.

Apart from the unexhausted recording of oral health behaviors, there are other limitations in this study. First, items about different tooth brushing techniques and types of toothbrush should be included in the assessment of tooth brushing behavior. Second, the caries status and periodontal status of adolescents need attention. It is meaningful to evaluate the relationship between the behavior change of adolescents and the long term improvements of it, which is reflected on caries status and periodontal condition in future studies. Third, our student sample represents a specific, narrow population in Hong Kong. Hence, this instrument should be tested in different populations to assess its generalizability. Fourth, it is not a longitudinal study; therefore, the model hypothesized from this study was not definitive. Fifth, the rate of using dental floss was low among Hong Kong adolescents [42]. The respondents might tend to perceive oral health behaviors as tooth brushing rather than flossing and resulted in an inaccurate answer about the oral health belief towards the concept. Last but not the least, the brushing and flossing frequencies were self-reported and were inherently biased by the social desirability of ideal answers. As a result, the negative effect of oral health behaviors on oral health might be underestimated.

## Conclusion

Overall, our findings strongly suggest that the final version of the 35-item scale based on the HBM is a valid and reliable instrument for measuring factors influencing oral health behaviors for adolescents. HBM variables can be used as predictors for oral health behaviors and oral health status. Additional research is recommended to evaluate the practicability, generalizability and applicability of this instrument to other populations.

## Supplementary information

**Additional file 1: Table S1.** The characteristics of participants.

**Additional file 2: Table S2.** Standardized bootstrapped direct effects with bias-corrected 95% CIs for final model of VPI.

## Data Availability

The datasets used and/or analyzed during the current study are available from the corresponding author on reasonable request.

## Abbreviations

HBM: Health belief model
VPI: Visual plaque index
KMO: KAISER-Mayer-Olkin
CFI: Comparative fit index
TLI: Tucker-Lewis index
RMSEA: Root mean square error of approximation
ICC: Correlation coefficients
SRMR: Standardized root mean square residual
EFA: Exploratory factor analysis
CFA: Confirmatory factor analysis
