# Supplementary material for: Development and validation of the Oral health behavior questionnaire for adolescents based on the health belief model (OHBQAHBM)
Source: BMC Public Health. 2020 May 15;20:701. doi: 10.1186/s12889-020-08851-x (PMC7227318; doi:10.1186/s12889-020-08851-x)
Supplement: Supplementary file 1 — Additional file 1: Table S1. The characteristics of participants. [file 12889_2020_8851_MOESM1_ESM.docx]

| Mean age years (SD) | 13.2 (0.5) |
| --- | --- |
| Sex, n (%) |  |
| Boys | 209 (49.9%) |
| Girls | 210 (50.1%) |
| Bushing behavior |  |
| Brushing less than twice a day | 81 (19.2%) |
| Brushing twice a day or more | 340 (80.8%) |
| Flossing behavior |  |
| Flossing less than once a week or never | 324 (77.0%) |
| Flossing once a week or more | 97 (23.0%) |
| Regular dental visit |  |
| Don’t have a regular dental visit plan | 277 (65.8%) |
| Have an annual dental visit | 144 (34.2%) |
| Perceived susceptibility subscale score | 2.6 (0.9) |
| Perceived benefits subscale score | 4.0 (0.6) |
| Perceived barriers subscale score | 2.1 (0.7) |
| Cues to action subscale score | 2.1 (0.9) |
| Perceived severity subscale score | 3.8 (0.8) |
| Self-efficacy subscale score | 3.6 (1.0) |
| VPI score | 2.3 (0.6) |

**Appendix Table 1. The characteristics of participants.**
