# Supplementary material for: Development and validation of the Oral health behavior questionnaire for adolescents based on the health belief model (OHBQAHBM)
Source: BMC Public Health. 2020 May 15;20:701. doi: 10.1186/s12889-020-08851-x (PMC7227318; doi:10.1186/s12889-020-08851-x)
Supplement: Supplementary file 2 — Additional file 2: Table S2. Standardized bootstrapped direct effects with bias-corrected 95% CIs for final model of VPI. [file 12889_2020_8851_MOESM2_ESM.docx]

| Significant effect on VPI score | β | Bootstrapped SE of β | Bias-Corrected 95% CI | P value |
| --- | --- | --- | --- | --- |
| Standardized direct effect | | | | |
| Self-efficacy | -0.105 | 0.046 | -0.201 to -0.026 | 0.015 |
| Flossing habit | -0.166 | 0.047 | -0.256 to -0.064 | 0.007 |
| Standardized indirect effect | | | | |
| Self-efficacy | -0.007 | 0.003 | -0.018 to -0.002 | 0.002 |
| Perceived severity | -0.005 | 0.003 | -0.012 to -0.002 | 0.001 |
| Cues to action | -0.016 | 0.011 | -0.043 to 0.001 | 0.056 |
| Perceived benefits | -0.007 | 0.004 | -0.016 to -0.002 | 0.003 |
| Perceived susceptibility | 0.025 | 0.012 | 0.007 to 0.056 | 0.004 |
| Perceived barriers | 0.031 | 0.012 | 0.010 to 0.063 | 0.003 |

**Appendix Table 2. Standardized bootstrapped direct effects with bias-corrected 95% CIs for final model of VPI**
